# *In –silico* molecular docking analysis of prodigiosin and cycloprodigiosin as COX-2 inhibitors

**DOI:** 10.1186/2193-1801-2-172

**Published:** 2013-04-19

**Authors:** Pabba Shiva Krishna, Kompally Vani, Metuku Ram Prasad, Burra Samatha, Nidadavolu Shesha Venkata Sathya Siva Surya Laxmi Hima Bindu, Maringanti Alaha Singara Charya, Prakasham Reddy Shetty

**Affiliations:** Department of Microbiology, Kakatiya University, Warangal, 506009 India; Department of Informatics Nizam College Hyderabad, Hyderabad, India; Department of Bioengineering & Environmental Center, Indian Institute of Chemical Technology, Tarnaka, Hyderabad, 500607 India

**Keywords:** Antiinflammation, COX-2, Cycloprodigiosin, Molecular docking, Prodigiosin

## Abstract

Prodigiosin and cycloprodigiosin are tripyrrole red pigmented compounds with medical importance for their anticancer property. In the present investigation, molecular docking studies were performed for both prodigiosin and cycloprodigiosins to evaluate the *in- silico* anti-inflammatory activity against Cycloxigenase-2 (COX-2) protein as model compound and the data compared with rofecoxib and celcoxid. Cycloprodigiosin showed higher initial potential, initial RMS gradient and potential energy values compared to prodigiosin. Analysis of COX-2 protein and ligand binding revealed that cyclprodigiosin interacted with COX-2 protein amino acid residues of Tyr^324^, Phe^487^ and Arg^89^ while prodigiosin interaction was observed with two amino acids i.e. Leu^321^ and Tyr^324^. The computational ligand binding interaction suggested > 45% higher fitness score value for prodigiosin to that of cycloprodigiosin with COX-2 protein while the standard compounds rofecoxib and celecoxid revealed fitness score of 44 and 62, respectively. The prodigiosin ligand revealed the best fitness score compared with the standard drug rofecoxib suggesting the prodigiosin could be effective as the potential inhibitor compound against COX-2 protein and can be evaluated as anti-inflammatory drug molecule using clinical trials.

**Electronic supplementary material:**

The online version of this article (doi:10.1186/2193-1801-2-172) contains supplementary material, which is available to authorized users.

## Introduction

Inflammation is the tissue reaction against infection, irritation or foreign substance. It is a part of the host defense mechanisms that is known to be involved in the inflammatory reactions associated with the release of histamine, bradykinin & prostaglandins. Clinically inflammation, reported by Cornelius Celsus of Rome 2000 years ago, is rubor (redness) or calor (heat) and /or dolar (pain) at the affected region (Chaudhary 2001) because of a complex biological response of vascular tissues to harmful stimuli including pathogens, irritants or damaged cells (Denko 1992).

Cyclooxygenases (COX) or prostaglandin endoperoxide synthases (PGHS) are the key enzymes in the synthesis of prostaglandins, the main mediators of inflammation, pain and increased body temperature (hyperpyrexia). The body produces two main isoforms COX proteins i.e., cyclooxygenases −1 (COX-1) and cyclooxygenases-2 (COX-2). The COX-1 is responsible for formation of important biological mediators such as prostanoids, including prostaglandins, prostacyclin and thromboxane and involved in pain causing, blood clotting and protecting the stomach (Watson et al. 2000) whereas COX-2 involved in the pain by inflammation and plays a major role in prostaglandin biosynthesis in inflammatory cells and central nervous system (Chhajed et al. 2010). When COX-1 is inhibited, inflammation is reduced, but the protection of the lining of the stomach is also lost. This can cause stomach upset as well as ulceration and bleeding from the stomach and even the intestines. Whereas, COX-2 is usually specific to inflamed tissue, there is much less gastric irritation associated with COX-2 inhibition together with the decreased risk of peptic ulceration (McGettigan & Henry 2006). Therefore, selective COX-2 inhibitors such as celecoxib and rofecoxib had been developed for ease of inflammation associated with COX (Hawkey 1999). The use of coxib drugs such as rofecoxib (Vioxx®) and valdecoxib (Bextra®) were withdrawn from the market in 2004 and 2005, respectively, because of increased risk of heart attacks and strokes with long term use (Mason et al. 2006). On the other hand, some studies have suggested that rofecoxib’s adverse cardiac events may not be a class effect but rather an intrinsic chemical property related to its metabolism (Hinz & Brune 2002). At present, Celecoxib (Celebrex®) is the only COX-2 inhibitor available in the United States. Hence, there is a need for COX-2 inhibitor with no adverse effects.

The development of non-steroidal drugs for inflammation especially in overcoming Rheumatoid arthritis has evoked much interest in the extensive search for new drugs with anti-inflamatory property (Dandiya & Kulkarni 1995). Prodigiosins, red pigment compounds produced by certain gram positive bacterial strains, gained pharmaceutical and human health care sector importance mainly due to their selective diverse biological activities mainly in inhibition of tumor derived cell lines proliferation with no apparent toxicity towards normal cells (Yamamoto et al. 1999; Liu et al. 2005) reported that the tumor cell proliferation inhibition is associated with the induction of apoptosis independent of p53 (Castillo-Avila et al. 2005) by suppressing the growth of tumor originated from chronic lymphocytic leukemia (Campàs et al. 2003) at metastasis (Zhang et al. 2005).

However, prodigiosins role in anti-inflammatory function is rarely reported. In the present investigation efforts have been made to evaluate prodigiosin and cycloprodigiosins, a secondary metabolite alkaloid with a unique tripyrrole chemical structure produced by a few species such as *Serratia, Pseudomonas* and *Streptomycin* (Song et al. 2006; Giri et al. 2004) anti-inflammatory function associated with COX-2 based on docking analysis as anti-inflammatory agent. This approach is adopted as evaluation of biological function of any compound especially associated with human trials which is a long term process and always risky. In this context, molecular docking continues to hold great promise in the field of computer based drug design, which screens small molecules by orienting and scoring them in the binding site of a protein as a result, novel ligands for receptors of known structure were designed and their interaction energies were calculated using the scoring functions. In view of the above, the present investigation merits in understanding the imperative role of prodigiosin and cycloprodigiosin anti-inflammatory properties against COX-2 protein based on fitness score, type of binding pattern, energy values etc.

## Materials and methods

### Protein preperation

The X-ray crystallographic structure of COX-2 (PDB ID 1cx2) protein was obtained from the Protein Data Bank at a resolution of 3.0Å. Water molecules, ligands and other hetero atoms were removed from the protein molecule along with the chain B, C and D. Addition of hydrogen atoms to the protein was performed using CHARMm force field. Energy minimization was performed by using conjugate gradient method with an RMS gradient of 0.01kcal/Å mol on Accelyrs Discovery studio client (version 2.5) software.

### Ligand preperation

**Figure 1:**
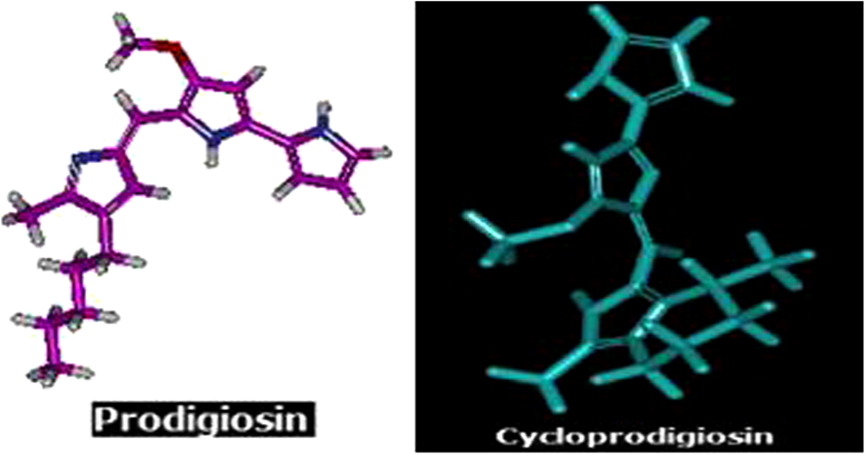
**3D structure of energy minimized ligand molecules.**

The ligand molecules (prodigiosin, cycloprodigiosin, celecoxib and rofecoxib) structure were drawn in Hyperchem molecular modeling and visualization tool (version 7.5) and the energy was minimized using Accelyrs Discovery studio client (version 2.5) software. The minimized protein and ligands were saved in PDB and mol-2 format, respectively for further analysis as shown in the Figure 1 and the energy values obtained were shown in Table 1.

**Table 1:** **Energy values of prodigiosin and cycloprodigiosin before and after energy minimization**

Parameter	Prodigiosin	Cycloprodigiosin
Initial potential energy	57.223	150.369
Initial RMS gradient	22.477	43.156
Potential energy	27.531	40.730
Vanderwaals energy	−7.284	−1.337
RMS gradient	0.107	0.0089

### Docking using GOLD

Docking simulations were performed using GOLD version 4.1.2 for the present study for predicting the protein–ligand interactions according to Selvaraj (Selvaraj & Malik 2008). GOLD uses genetic algorithm for docking and performs automated docking with fully cyclic ligand flexibility, partial cyclic ligand flexibility and partial flexibility in the neighborhood of the protein active site (Spassov et al. 2008). The docking process involves a conformational search for compound which compliments a target binding site, with the aim of identifying the best matching pose (Chitra & Jeyanthi 2011) along with the active site to perform docking. The stability of docked ligand-protein complex is due to hydrogen bonding and Vanderwaals interactions.

The energy minimized protein and ligand along with the binding site atom number or the X, Y and Z points of the Nitrogen atom of the any of the binding site residue submitted to the GOLD setup. All the atoms within 10 Å of the given binding residue atom number were selected for binding pocket. The default parameters of the automatic settings were used to set the genetic algorithm parameters. The docked conformation which had the highest Gold Score was selected to analyze the mode of binding.

The gold score (fitness), energy, bond and vanderwaals energies are visualized in gold report which is used for further analysis. The gold score is a molecular mechanics like function with four terms S(hb_ext), S(vdw_ext), S(hb_int) and S(int).

## Result and discussion

In the present investigation, to screen out potential of anti-inflammatory properties of the selected tripyrrole compounds (prodigiosin and cycloprodigiosin) were evaluated through GOLD 4.1 molecular docking studies by using *in silico* analysis. Initially, the 3D ligands of these molecules were generated (Figure 1) followed by energy minimization. The obtained energy minimization values of selected prodigiosin and cycloprodigiosin were reported in Table 1. It was noticed that cycloprodigiosin has higher initial potential, initial RMS gradient and potential energy values compared to prodigiosin (Table 1). The variation in these energy values observed to be different which is apparent due to the structural difference between these natural pigments of same class. This can be exemplified from the fact that initial potential energy value for cycloprodigiosin was approximately three-fold while initial RMS gradient and potential energy values were more or less two-fold to that of prodigiosin. Further, vanderwaals energy value of prodiogiosin was seven-fold lower compared to cycloprodigiosin. Such lower vanderwaals energy value denoted the impact of hydrogen bonding property of these compounds during protein/enzyme interaction.

**Figure 2:**
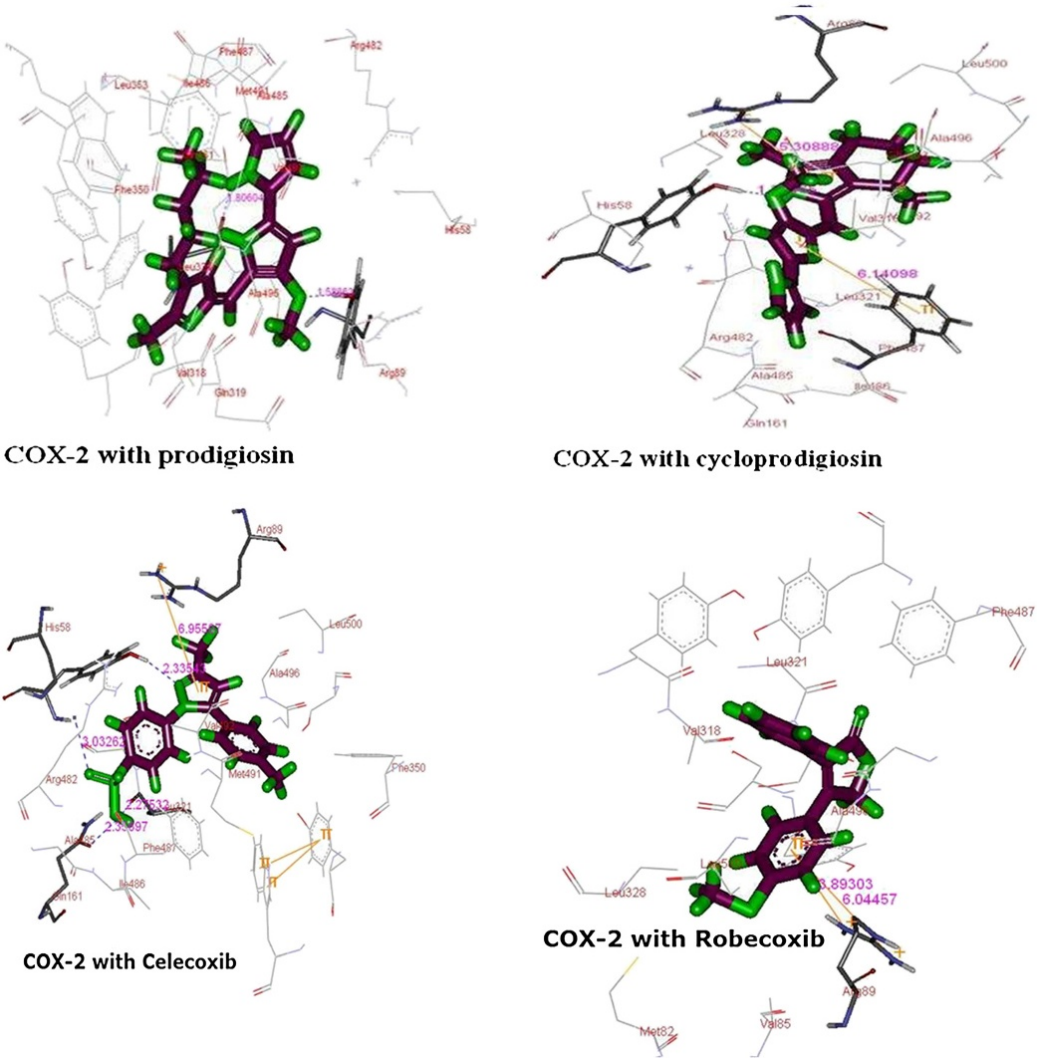
**Binding pose of ligand molecules of selected (prodigiosin and cycloprodigiosin) and standard (cellecoxib and rofecoxib) with different COX-2 protein (Docking of ligand into the binding pocket of inflammatory proteins establishing interactions with the active site default colors).**

Structure-functional relationship of prodigiosin and cycloprodigiosin was evaluated to know their biological activity against the COX-2 using the 3D structure of the receptor retrieved from protein data bank site of COX-2 enzyme (pdb code: COX-2). For this the docked binding mode was established to link the docking scoring function with these selected compounds and protein. Analysis of the binding pattern between COX-2 protein and ligand suggested that the binding pattern also varied with the ligand nature (Figure 2). This could be exemplified based on the observation that cyclprodigiosin interacted with COX-2 protein amino acid residues of Tyr^324^, Phe^487^ and Arg^89^ while prodigiosin interaction was observed with only two amino acid residues i.e., with Leu^321^ and Tyr^324^. However, the interaction of standard anticancer compound, rofecoxib, was noticed with only one amino acid residue i.e., Arg^89^ of COX-2 protein, whereas other standard compound, celecoxib, indicated binding pattern with five amino acid residues (His^58^, tyr^324^, gln^161^, leu^321^ and Arg^89^) (Table 2). This docking data with COX-2 protein active site amino acid residues revealed that these two selected prodigiosin and cycloprodigiosin interact with COX-2 protein other than active site. This is because, it was well documented in the literature that COX-2 active possess three important regions; a hydrophobic pocket characterized by the presence of Tyr^385^, Trp^387^, Phe^518^, Ala^201^, Tyr^248^ and Leu^352^. The second key region is associated with three hydrophilic amino acid residues (Arg^120^, Glu^524^ and Tyr^355^) which is located at the entrance of the active site while third is a side pocket characterized with the presence of His^90^, Arg^513^ and Val^523^ (Priscilla et al. 2011). The obtained docking data is in accordance with reported data on synthetic compounds where amino acid residues such as His^90^, Arg^120^, Gln^192^, Val^349^, Leu^352^, Ser^353^, Tyr^355^, Leu^359^, Tyr^385^, Trp^387^, Arg^513^, Ala^516^, Phe^518^, Val^523^, Gly^526^, Ala^527^, Leu^531^ associated with A chain of COX-2 protein were involved for protein–ligand complementarily activity.

**Table 2:** **Type of interactions and interacting amino acid residues of COX-2 protein with selected ligands**

COX-2 Protein	H-Bonding	V-waal	Pi-pi	Pi-sigma	Pi-cation
Cycloprodigiosin	Tyr^324^	-----	Phe^487^	-----	Arg^89^
Prodigiosin	Leu^321^, Tyr^324^	-----	-----	-----	-----
Rofecoxib	-----	-----	-----	-----	Arg^89^
Celecoxib	His^58^, tyr^324^, gln^161^, leu^321^	-----	-----	-----	Arg^89^

Critical evaluation of the nature of binding interaction of these selective prodigiosin and cycloprodigiosin further indicated that H-bonding with two amino acid residue, Leu^321^ and Try^324^ of COX-2 protein is the only one associated with prodigiosin while in case of cycloprodigiosin the binding is by three different interactions; H-bonding with Tyr^324^, Pi-Pi interaction with Phe^487^ and Pi-Cation nature of binding with amino acid residue Arg^89^ of COX-2 protein (Table 2). This binding pattern data further suggested that H-binding is common with both selected tripyrrole compounds i.e. prodigiosin and cycloprodigiosin (Table 2). The observed anti-inflammatory activity with rofecoxib and celecoxib in association with interactive binding with COX-2 protein further denote that the selected prodigiosin and cycloprodigiosin could be effectively used as anti- inflammatory agents. This could be confirmed based on the fact that both referral compounds (rofecoxib and celecoxib) showed Pi-cation interaction with COX-2 protein at Arg^89^ (Table 2). In addition, H-bonding also observed in case of celecoxib with four different amino acid residues (His^58^, Tyr^324^, Gln^161^ and Leu^321^) of COX-2 protein. It is interesting to note that prodigiosin, cycloprodigiosin and celecoxib have common binding site at Tyr^324^ of COX-2 protein. Further, celecoxib also observed to reveal interaction with COX-2 protein which is similar to that of H-bonding of prodigiosin with Leu^321^ and cycloprodigiosin Pi-cation interaction with Arg^89^ residue. This observation further confirm that selected prodigiosin and cycloprodigiosin may be an effective anti-inflammatory compounds especially with respect to COX-2 protein mediated inflammation however, differ in bonding pattern with protein (Dilber et al. 2008; Llorens et al. 2002). This could be also evidenced from the energy minimized 3D structures of prodigiosin and cycloprodigiosin (Figure 1). This data is contradictory with literature reports where docking of the synthetic compounds depicted three different types of binding patterns in general. Use of selective COX-2 inhibitors such as SC-558, the bonding was in the close vicinity of the hydrophobic pocket and the phenylsulphonamide group occupied the side pocket and showed binding with His^90^ and an interaction with Arg^513^ which has also been identified as an important residue in the binding of selective COX-2 inhibitors according to the site- directed mutagenesis data (Kurumbail et al. 1996). However, in another study, docking of Diclofenac revealed that its orientation makes the residues of side pocket thereby the hydrophilic pocket of COX-2 protein is inaccessible and the phenyl acetic acid moiety is orientated towards Tyr^385^ and Ser^530^ and hence possess H-bonding interaction (Dilber et al. 2008). Ibuprofen and Naproxen when docked into the active site of the COX-2 enzyme, the interacting residues 120 were observed to be Arg^120^ and Tyr^355^ (Llorens et al. 2002). This comparative analysis of literature data and present investigation further indicated that the prodigiosin and cycloprodigiosin influence the active site confirmation of COX-2 protein by interacting at different place other than active site and induces the anti-inflammatory function.

In view of the above, fitness score values were measured using Swiss PDB viewer considering steric and electrostatic properties. The data revealed binding pattern differ with the 3D topology of the prodigiosin to cycloprodigiosin and influence fitness score value. Higher fitness score of 59 was noticed with prodigiosin while only fitness score of 37 was observed for cycloprodigiosin (Table 3) suggesting that more interaction of prodigiosin with COX-2 enzyme. This is interesting because, though higher fitness score was noticed for prodigiosin, its interaction with COX-2 protein was only with two amino acid (Leu^321^ and Tyr^324^) residues (Table 2) while, cycloprodiogiosin however, showed interaction with three amino acid (Tyr^324^, Phe^487^ and Arg^89^) residues (Table 2). Further analysis of external hydrogen binding pattern between enzyme and selected tripyrrole compounds denoted 1.95 and 1.62 for prodigiosin and cycloprodigiosin, respectively. However, interaction between enzyme and compounds was not observed at internal hydrogen bonding level (Table 3). In addition, evaluation of vanderwalls interaction denoted more than 20% higher bonding with prodigiosin to that cycloprodigiosin (Table 3). The Table 1 also indicated the static interaction relationship between COX-2 and selected tripyrrole compounds. The observed negative interaction values do indicate a better steric interaction. It is evident that more steric interaction was noticed with cycloprodigiosin compared to prodigiosin. Based on docking, prodigiosin could be a potential anti-inflammatory agent against COX-2 associated inflammation reactions.

**Table 3:** **Fitness score values as well as hydrogen bonding interaction values between COX-2 protein and ligand molecules**

COX-2 protein	Fitness	S(hb_ext)	S(vdw_ext)	S(hb_int)	S(int)
Prodigiosin	59.62	1.95	48.88	0.00	−9.54
Cycloprodigiosin	37.61	1.62	37.42	0.00	−15.46
Rofecoxib	44.59	0.00	33.65	0.00	−1.69
Celecoxib	62.15	2.18	46.59	0.00	−4.09

## Conclusions

The development of novel compounds with biological activity is an urgent need. In the present study the COX-2 protein was successfully docked onto the both prodigiosin and cycloprodigiosin for drug interaction study to have a track in the ongoing race between drug development and new drugs especially new compounds which are more important for the discovery of new hits using molecular methods. The Fitness scores of prodigiosin and cycloprodigiosin were calculated using the GOLD software. Though the binding pattern of ligands with COX-2 differed respect to H-bonding, Pi- interaction and Pi-cation of prodigiosin and cycloprodigiosin, fitness score values substantiate the hypothesis that prodigiosin has the potential to inhibit the COX-2 protein.

Hence, it is concluded that that prodigiosin could be a potent antinflamatory target molecule against COX-2 which may be worth for further clinical trails.

## Authors’ original submitted files for images

Below are the links to the authors’ original submitted files for images. Authors’ original file for figure 1 Authors’ original file for figure 2 Authors’ original file for figure 3
