# Supplementary figures and images for: In –silico molecular docking analysis of prodigiosin and cycloprodigiosin as COX-2 inhibitors
Source: Springerplus. 2013 Apr 19;2(1):172. doi: 10.1186/2193-1801-2-172 (PMC3667375; doi:10.1186/2193-1801-2-172)

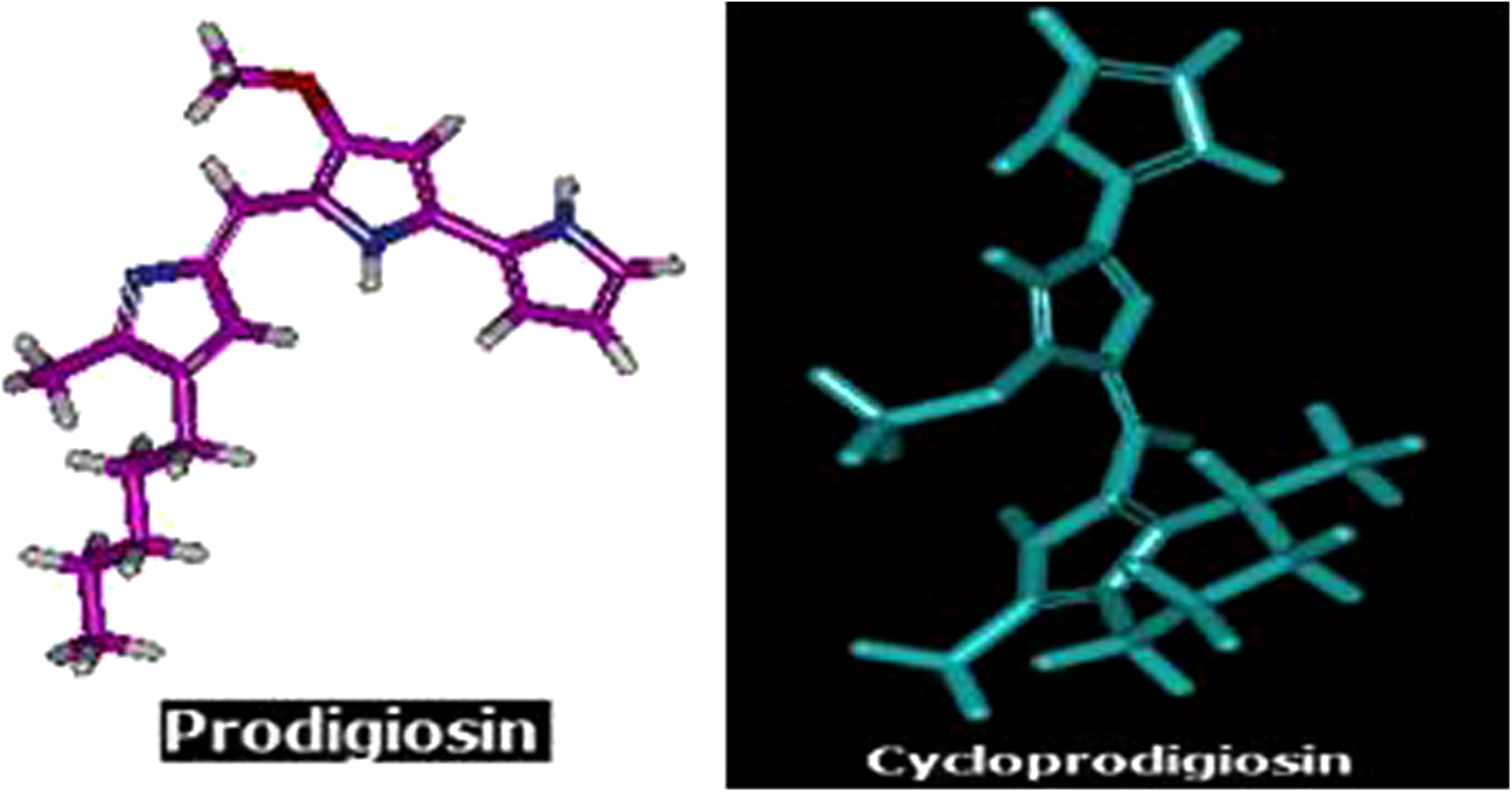

Supplement: Supplementary file 1 — Authors’ original file for figure 1 [file 40064_2013_286_MOESM1_ESM.tiff]

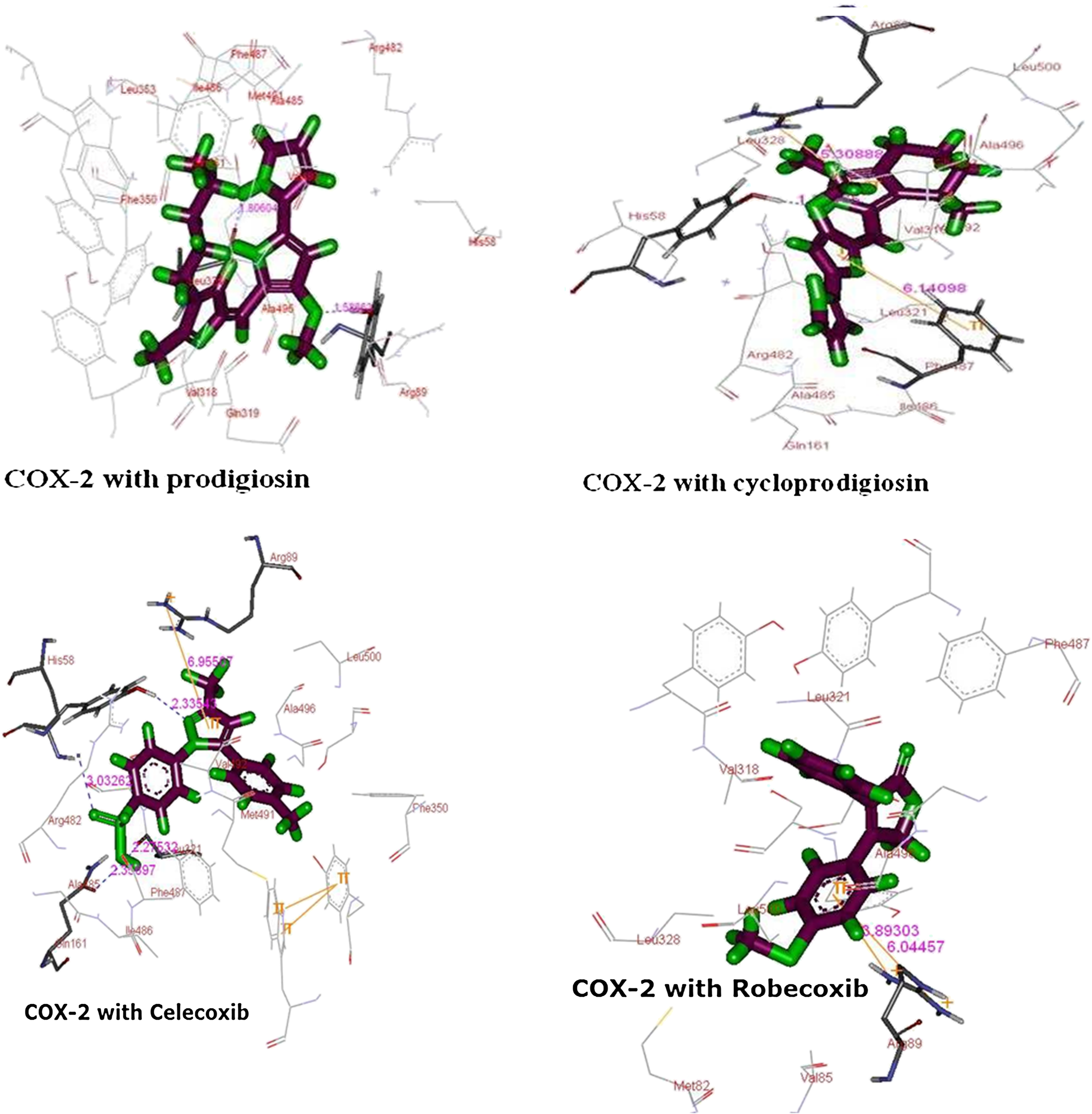

Supplement: Supplementary file 2 — Authors’ original file for figure 2 [file 40064_2013_286_MOESM2_ESM.tiff]

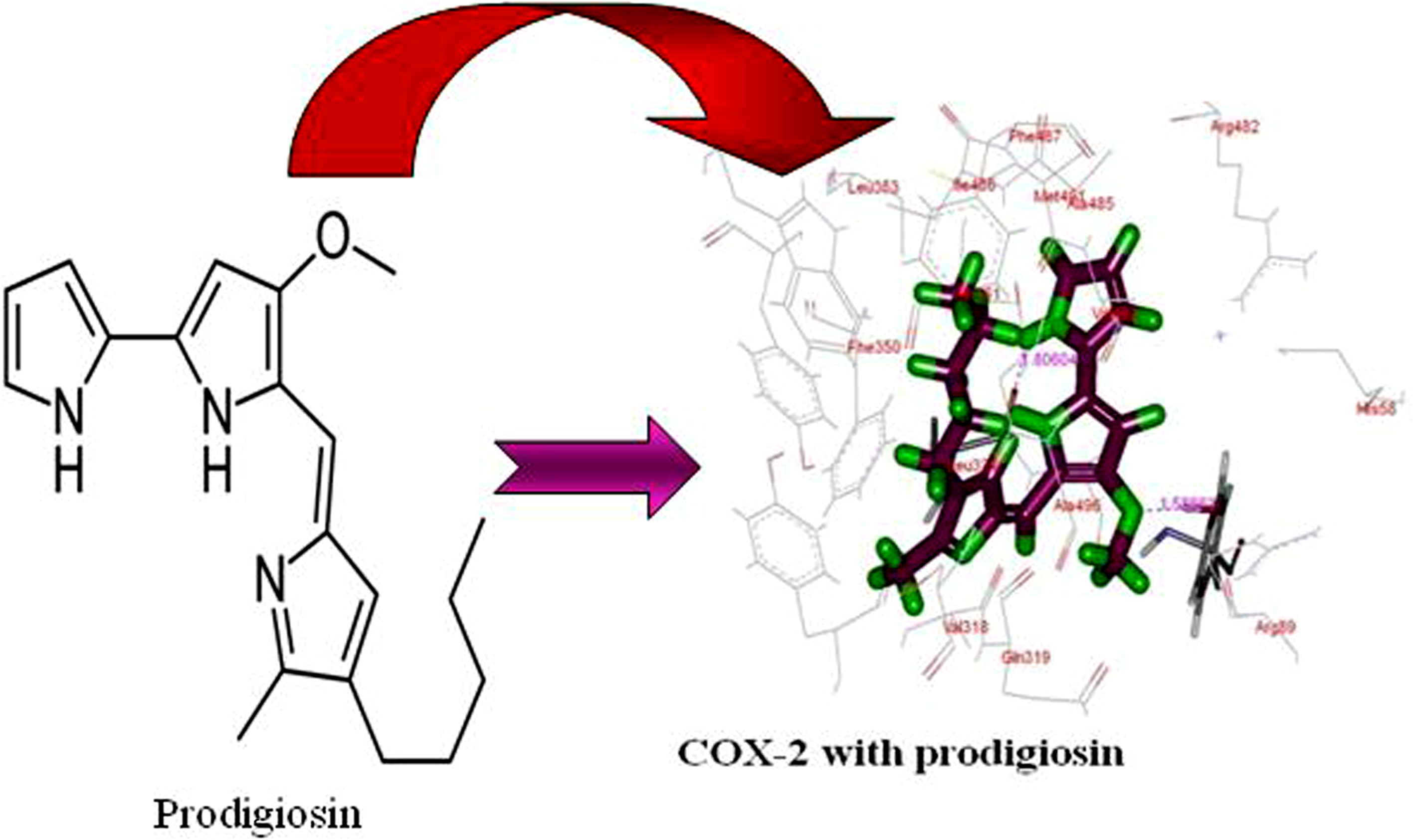

Supplement: Supplementary file 3 — Authors’ original file for figure 3 [file 40064_2013_286_MOESM3_ESM.tiff]
